# The development of HTA collaboration at European level and the challenge of the EU regulation

**DOI:** 10.1093/eurpub/ckac129.086

**Published:** 2022-10-25

**Authors:** A Ruether, E Petelos

**Affiliations:** 1 IQWiG, Köln, Germany; 2 Health Services Research, CAPHRI, Maastricht, Netherlands; 3 Clinic of Social and Family Medicine, University of Crete, Iraklion, Greece

## Abstract

**Issue:**

The establishment of HTA has been an important topic in Europe for many years. The engagement of HTA agencies, scientists, politicians and many other stakeholders throughout more than 20 years lead to the EU-HTA regulation, which came into force beginning of 2022. Now it is time to prepare for a sustainable European Network for HTA.

**Description:**

The implementation of HTA in Europe is of high importance of the EU-commission for more than 20 years. Started with the first projects in 1994 (EUR-ASSESS) it needed quite a bunch of different projects and, in the end, three joint actions for finally providing legal ground for HTA with the EU-HTA regulation. This journey seemed a long one. However, referring to the need of addressing the requirements of Health Care of 27 Member States combined with the demand of reliable high methodological quality and applicability, investing all the time and effort for a sustainable European Network of HTA was definitely worthwhile. Joint Scientific Consultation (JSC) and Joint Clinical Assessments (JCA) will play a central role for national HTA and decision making in health care. But for understanding role, content, national uptake and further development of means, products and results of a European Network for HTA it is quite important to get a perception of the development of HTA collaboration in Europe. Based on this the challenge of the EU-HTA regulation and its implementation will be tackled easier.

**Effects:**

Through provision of the main pillars of the previous EU-HTA collaboration the appreciation and understanding of the differences and complexities behind the HTA processes in the EU-healthcare systems will made aware. Based on this understanding the challenges of the implementation of the EU-HTA regulation will be discussed.

